# The Platelet Function Analyzer (PFA-100) as a Screening Tool in Neurosurgery

**DOI:** 10.5402/2012/839242

**Published:** 2012-08-08

**Authors:** Ralf Karger, Karoline Reuter, Jochen Rohlfs, Christopher Nimsky, Ulrich Sure, Volker Kretschmer

**Affiliations:** ^1^Medizinische Fakultät, Philipps-Universität Marburg, Conradistraße, 35043 Marburg, Germany; ^2^Praxis für Transfusionsmedizin, Aachener Straße 313, 50931 Köln, Germany; ^3^Klinik für Neurochirurgie, Philipps-Universität Marburg, Conradistraße, 35043 Marburg, Germany; ^4^Klinik für Neurochirurgie, Universitätsklinikum Essen, Hufelandstraße 55, 45147 Essen, Germany

## Abstract

We investigated whether the inclusion of the PFA-100 in the preoperative screening of neurosurgical patients might reduce perioperative bleeding complications. Patients with intracranial space-occupying lesions who were scheduled for neurosurgery underwent routine preoperative PFA-100 testing. In case of an abnormal PFA test, patients received prophylactic treatment with desmopressin. 93 consecutive patients were compared to 102 consecutive patients with comparable characteristics operated before introduction of the PFA-100 testing. 2 patients (2.2%) in the PFA group and 2 patients (2.0%) in the non-PFA group experienced clinically relevant intracranial bleeding confirmed by computed tomography (OR 1.05, 95% CI 0.39–2.82; *P* = 1.0). Transfusions were not significantly different between the two groups. 13 (14.0%) patients in the PFA group and 5 (4.9%) patients in the non-PFA group received desmopressin (OR 3.2, 95% CI 1.1–9.2; *P* = 0.045). Preoperative screening with the PFA-100 did result in a significant increase in the administration of desmopressin, which could not reduce perioperative bleeding complications or transfusions.

## 1. Introduction

Hemorrhage is one of the most threatening and feared complications of surgery. In neurosurgery, in particular, hemorrhage can lead to devastating consequences for the patient [1–3] because of the limited possibility of spontaneous drainage of the blood and the impairment of the surgeon's view.

In addition, perioperative hemorrhage often requires the administration of blood products in order to replenish a deficit of red cells and/or to correct resulting or underlying coagulopathy. The risks of blood transfusion still cause significant morbidity and mortality [4], and, in addition, the gap between the supply of blood products and the ever-increasing demand is widening. It is anticipated that, because of the demographic changes in industrialized countries [5], already in the near future the postponement or even cancellation of interventional procedures due to the lack of an adequate blood supply might become a common scenario.

In recent years it has become apparent that a significant number of patients scheduled for surgery exhibit disorders of hemostasis, which might increase the risk of perioperative bleeding. It has been shown that disorders of primary hemostasis are much more prevalent in these patients than disorders of secondary hemostasis [6, 7]. Only the latter can be, although not with adequate certainty [8], detected by common preoperative screening tests of hemostasis, for example, prothrombin time (PT) and activated partial thromboplastin time (aPTT). Many patients take antiplatelet or nonsteroidal anti-inflammatory drugs without disclosing this upon the preanesthetic visit. A plethora of other medications can impair platelet function to a clinically significant degree (e.g., serotonin reuptake inhibitors, antibiotics, and volume expanders) [9]. Thus, it is expected that, in a significant number of neurosurgical patients, clinically significant disorders of primary hemostasis remain unnoticed, eventually leading to preventable bleeding complications.

For decades, the bleeding time used to be the only diagnostic tool to evaluate primary hemostasis. However, this test is cumbersome and difficult to standardize and, consequently, has shown only very limited value in correctly predicting clinically significant disorders of primary hemostasis [10]. Over the last 20 years, attempts to develop an easy-to-use and reliable *in vitro* tool for investigating primary hemostasis [11] resulted in the launch of the Platelet Function Analyzer (PFA-100). Although, with this instrument, one cannot investigate the vascular component of primary hemostasis, it has been shown to be useful in a variety of clinical situations [7, 12–14].

A large body of evidence has accumulated that disorders of primary hemostasis can often successfully be treated with desmopressin (1-deamino-8-D-arginine-vasopressin (DDAVP)). Desmopressin is a selective vasopressin receptor type 2 agonist and leads to the release of von-Willebrand factor from the endothelium into the circulation. Apart from its role in von-Willebrand disease [15] desmopressin has been shown to be effective in a number of congenital, acquired and drug-induced platelet function defects [16]. On the other hand, it has been demonstrated that the unselective preoperative use of desmopressin does not reduce blood loss and transfusion requirements in cardiac, vascular, or orthopedic surgery patients [17]. Desmopressin appears to be an effective hemostatic agent only when a disorder of primary hemostasis is evident. The use of desmopressin in neurosurgery to treat platelet function defects has been suggested [18, 19] but no reports applying this approach systematically have been published so far.

We assumed that, by the introduction of the PFA-100 in the routine preoperative evaluation of elective patients with intracranial space-occupying lesions who were scheduled for complex neurosurgical interventions, we could prevent bleeding complications and reduce blood product consumption by administering desmopressin to patients where an abnormal preoperative PFA-100 result was found. It was the aim of this study to analyze whether this approach indeed produced the presumed beneficial effects.

## 2. Patients and Methods

### 2.1. Patients

The PFA-100 was introduced in April 2005 into the routine preoperative screening of neurosurgery patients after a decision of a clinical board consisting of the heads and deputy heads of the Department of Transfusion Medicine and Hemostaseology and the Department of Neurosurgery. It was concluded that elective patients with intracranial space-occupying lesions who were scheduled for complex neurosurgical interventions were most likely to benefit from this approach, and thus PFA-100 testing was restricted to those patients. In addition, patients suffering from vascular malformations or intracranial aneurysms were excluded. This decision was based on the notion that in these patients bleeding is primarily a result of the intracranial pathology and the concern that certain side effects of desmopressin, that is, a possible rise in blood pressure or vasoconstriction in some cases, might be a risk for these patients that should not be taken.

Patients were consecutively recruited for analysis from a fixed time period of nine months after the introduction of the PFA-100 (PFA-group). They were compared to all patients meeting the clinical criteria described above that had been operated by the same team of surgeons and the same surgical techniques in the nine months before the introduction of the PFA-100 (non-PFA group). Patients operated on in April 2005 were excluded because we assumed the new regime needed some time to be adequately established in the participating departments. We preferred fixed time frames rather than identical patient numbers for the composition of both groups because we considered this approach less prone to a selection bias.

All patients were asked for previous bleeding problems upon the routine preoperative workup. Patients with an abnormal result in one or both of the commercially available PFA test modifications (see Section 2.2) on the day of routine preoperative evaluation received 0.3 *μ*g per kg of body weight desmopressin intravenously over 30 min beginning 90 min prior to surgery. Based on clinical judgment the infusion could be repeated intra- or postoperatively.

The approval of the institutional review board was not assumed necessary for this retrospective study because the intervention analyzed involved a test system that had been approved officially for clinical use and was introduced as a routine practice based on current scientific evidence. The study was only performed thereafter relying on routinely collected patient data.

### 2.2. Methods

The principle of the PFA-100 has been described previously [20]. In short, the system consists of a test cartridge, containing a capillary of 200 *μ*m diameter through which whole blood is drawn by applying negative pressure to the system. The test simulates primary hemostasis through interaction of platelets with the aperture of a membrane at one end of the capillary which is coated with collagen and either adenosine diphosphate (PFA-ADP) or epinephrine (PFA-Epi). The closure time (CT) of the aperture through adhesion and aggregation of the platelets is measured and is considered to represent primary hemostasis. The PFA-100 has been proposed for detection of impaired primary hemostasis in thrombocytopathic disorders, thrombocytopenia, von-Willebrand disease (vWD), and in patients taking aspirin [21, 22]. The CT is also influenced by the hematocrit [23], which might be of clinical relevance [24]. Thus, the test is supposed to provide a global picture of nonvascular primary hemostasis that might be more clinically informative for the assessment of the bleeding risk than tests evaluating single components of primary hemostasis because compensatory mechanisms can be taken into account. However, particularly in thrombocytopenia, as well as in certain thrombocytopathic disorders, or in patients taking drugs other than aspirin in order to suppress platelet function, test results are inconsistent and sometimes difficult to interpret.

Patient baseline data were retrieved from the routine hospital patient records. All patients that had undergone neurosurgery during the study period were initially evaluated. Patient selection was performed by two authors (K. Reuter and J. Rohlfs) by mutual agreement. Patient selection was based on initial diagnosis and the *WHO Classification of Tumours of the Nervous System* [25]. Surgical experience was newly assessed for this study as a parameter that might influence outcome and thus could have caused a different prognosis of the groups at baseline. With respect to level of training, surgeons were categorized as most experienced (level 3), experienced (level 2), and less experienced (level 1). There were cases where categorization was not possible, for example, when the surgeon was replaced during surgery. 

The following outcome data were retrieved from the patient records: Glasgow Coma Scale (GCS) [26], Glasgow Outcome Score (GOS) [27], intraoperative bleeding tendency (as routinely assessed by the surgeon and, if abnormal, documented in the surgical report), duration of anesthesia and surgery, hemostatic treatment (apart from transfusions, see below), and length of stay in intensive care. Bleeding complications were newly assessed for this study on routinely available postoperative computed tomography (CT) scans in a blinded fashion by one author (J. Rohlfs). A bleeding complication was classified as clinically relevant if at least one of the following criteria was satisfied:

- cerebrospinal fluid block,
- midline deviation,
- intraventricular bleeding,
- considerable deterioration of consciousness together with CT-documented bleeding but none of the above.

Transfusion requirements (including plasma derivatives) had been centrally documented in an electronic data processing system.

### 2.3. Statistical Analysis

The statistical analysis was performed with the statistic analysis software program SAS version 9.1 (SAS Institute, Cary, NC). Parametric variables were compared using the *t*-test. These data are presented as mean and standard deviation (SD). For nonparametric variables Wilcoxon's rank sum test was used. These data are presented as median and highest and lowest value (Min-Max). Categorical data were compared using the Chi-square test or Fisher's exact test depending on the numbers in the underlying contingency tables. For categorical outcome data, odds ratios with 95% confidence intervals (95% CI) were calculated, if necessary with adjustments for significantly different baseline data. *P* values are reported two-sided and were considered statistically significant if they were <0.05. No adjustments for multiple comparisons were made, since this was an exploratory analysis.

## 3. Results

### 3.1. Patient Demographics

Finally, 102 and 93 patients were included in the non-PFA group and the PFA group, respectively. Baseline characteristics are listed in Table 1. Important preoperative laboratory values are shown in Table 2. None of the analyzed parameters were significantly different between the two groups. However, there was a trend that the more experienced surgeons operated in the non-PFA group. The number of patients operated on by most experienced/experienced/less experienced surgeons was 30/52/14 (31.3/53.2/14.6%) and 16/58/16 (17.8/64.4/17.8%) in the non-PFA and PFA group, respectively (*P* = 0.10). The distribution of the pathological entities for both groups is shown in Table 3. Again, no statistically significant difference between the groups can be discerned.

### 3.2. Intervention

In 15 (16.1%) out of the 93 patients in the PFA group, an abnormal PFA result was found (*n* = 3 both tests, *n* = 10 PFA-Epi only, *n* = 2 PFA-Epi only with a PFA-ADP at the upper reference range). Only 12 of these 15 patients received desmopressin, two of whom also received aprotinin intraoperatively. In five of the 15 patients with an abnormal PFA result an intraoperative bleeding tendency was observed, even though four of them had received desmopressin preoperatively. Two of the 15 patients had a bleeding complication, one of which was categorized as clinically relevant. No preoperative desmopressin administration was documented for this patient. In the remaining 78 patients with a normal PFA test result, an intraoperative bleeding tendency or a clinically relevant bleeding complication was observed in one patient each. In this subgroup, desmopressin had been administered to one patient, aprotinin to three patients.

### 3.3. Outcome

Patient outcomes are listed in Tables 4 and 5. Postoperative hemoglobin concentration, duration of anesthesia and surgery, number of transfusions, and parameters of the postoperative clinical condition of the patient (GCS and GOS) were not significantly different between the groups. The risks to be transfused, to exhibit an intraoperative bleeding tendency, to suffer from a clinically relevant bleeding complication, or to receive the hemostatic agents aprotinin or tranexamic acid were also not significantly different between the groups. The patients in the PFA group were significantly more likely to receive desmopressin (*P* = 0.045).

## 4. Discussion

Diagnostic tools, in order to inform clinical management, need to be evaluated with respect to their influence on patient outcome. If a change in clinical management driven by the result of a diagnostic procedure does not improve patient outcome, this diagnostic procedure only produces additional costs and should be abandoned. It was the aim of this study to analyze the diagnostic utility of the PFA-100 for the improvement of the postoperative outcome of a clearly defined subgroup of neurosurgical patients. It could be shown that introduction of the PFA-100 into the routine of preoperative screening of elective patients with space-occupying lesions undergoing complex neurosurgical procedures did not result in an improved patient outcome. Several aspects of patient outcome had been studied, and none was significantly different between patients with and without preoperative PFA testing. There was not even a trend for a favorable outcome in any of the analyzed parameters for the patients in the PFA group. However, the patients in the PFA group received more often desmopressin than patients in the non-PFA group but obviously did not benefit from this treatment. Thus, this treatment must be regarded as ineffective in this situation, therefore unnecessarily subjecting these patients to potential side effects of the drug. Furthermore, it has been shown in experimental settings that vasopressin receptors play a role in the development of posttraumatic brain injury, even though type 2 receptors appear not to be involved in this process so far [28, 29]. It is conceivable, though, that desmopressin might also have a direct impact on the success of surgery.

However, previous studies have shown that disorders of primary hemostasis, and platelet function in particular, are common among patients undergoing surgery [6, 7, 9] and these disorders can often successfully be treated with desmopressin [16]. How can these promising clinical data be reconciled with the disappointing results of the present study? In order solve this conundrum several aspects need to be addressed. First, how often does the PFA-100 detect a clinically relevant platelet function defect, that is, to what extent is this defect responsible for the bleeding complication occurring during or after surgery? Second, how effective can desmopressin expected to be in these patients? Of course, the answer to the second question relies heavily on the answer to the first one. 

Two-thirds of the patients in this study displayed only an abnormal PFA-Epi test. The PFA-Epi is very sensitive to the effect of aspirin and can still be highly abnormal several days after ingestion of the last aspirin dose [30]. So, it is quite likely that many of these patients had undisclosed aspirin intake prior to surgery. However, the clinical impact of current aspirin ingestion is modest at best and does not invariably result in increased perioperative bleeding [31–34]. Thus, an abnormal PFA result can only indicate an increased risk for bleeding in this situation. It does not predict bleeding complications or transfusions in a deterministic way.

Moreover, perioperative bleeding is often caused by factors that cannot be anticipated preoperatively. One factor is obviously the risk inherent in the difficulty and complexity of the surgical procedure itself. In addition, coagulation disturbances that usually do not prevail or cannot be detected preoperatively (like increased fibrinolysis [35]) can play a significant role. Since desmopressin slightly induces fibrinolysis, the combined fibrinolytic effect of tumor pathophysiology and desmopressin could well have neutralized the hemostatic effect of desmopressin in some cases.

In summary, and to answer the second question, most of the preoperatively detected disorders of primary hemostasis in the study patients appear not to have been clinically relevant. In addition, other factors obviously have influenced bleeding complications and transfusion requirements. This may be exemplified by the fact that one of the patients in the PFA group with a bleeding complication did not have an abnormal PFA-test. This patient may of course have been suffered from a platelet defect not detectable by PFA-testing, which still is to be considered a failure of the analyzed management approach.

The foregoing considerations may explain why there was not a noticeable beneficial effect of desmopressin. It seems that the hemostatic balance and the clinical circumstances of the patients studied are far too complex as to allow a simple one-dimensional approach to be widely successful. 

Finally, the PFA-100 still has limited performance characteristics with respect to the diagnosis of clinically relevant disorders of primary hemostasis [13]. Diagnostic performance is especially compromised in patients with platelet secretion defects (in particular affecting the PFA-ADP) and in patients with low hemoglobin values [36]. This might also have precluded more favourable results for the investigated preoperative workup approach.

Our study undoubtedly bears one major limitation. The retrospective design is prone to impaired data quality and variables important for patient selection or outcome determination might not have been documented at all. In addition, by selection of a historical control group comparison is always susceptible to time effects that can mask or counteract effects attributable to the intervention studied. We tried to address these issues in several ways. Since PFA screening was introduced for a clearly defined patient group, selection of the control group was therefore quite straightforward and left little room for any subjective influence. This assumption is supported by the fact that both patient groups were highly comparable with respect to all analyzed baseline variables. It is hardly conceivable that any important baseline data with a significant impact on the study outcomes had been missed or would have been different between the groups. We tried to control any time effects by analyzing as short a study period as possible. A period of no more than in total 1.5 years was considered justifiable because data were eagerly needed in order to make a substantiated decision whether to continue or to give up this clinical approach. Furthermore, time effects owing to continuous but not always recognizable improvements in health care usually favor better outcomes of the patients treated later, that is, the PFA group in our case. However, since no better outcome of the patients in the PFA group was observed, this even strengthens the notion that there indeed seems to be no beneficial effect of routine preoperative PFA testing in the patient group studied. Thus, notwithstanding the shortcomings of our study design, we are convinced that the conclusions drawn from the data at hand are based on substantial evidence and can serve as a starting point for future investigations of this topic. However, given the low number of bleeding complications in this study, randomized trials designed to evaluate measures aimed at reducing the perioperative bleeding complication rates may be difficult to perform. The statistical proof of even a relative risk reduction of 50%, that is, from a 2% to a 1% bleeding complication rate, would need nearly 2,000 study participants per group.

## 5. Conclusion

This is the first study analyzing a systematic management approach to reduce perioperative bleeding complications and transfusion requirements in a cohort of vulnerable neurosurgical patients. According to our data, the correction of a platelet function deficit determined by a commercially available test of platelet function could not reduce bleeding complications and transfusion requirements but resulted in increased administration of desmopressin, a hemostatic drug which itself has potential unfavorable side effects that might compromise patient outcome. Further studies are needed to determine which patients may actually benefit from routine preoperative testing of platelet function and how this testing has to be tailored in order to detect only clinically significant defects. 

## Figures and Tables

**Table 1:** Patients clinical baseline characteristics.

Parameter	Non-PFA group	PFA group	*P* value
Age [Years]	50 (16)	50 (18)	0.91
Gender: male/female [*n*]	54/48	45/48	0.57
Height [cm]	172 (10)	172 (10)	0.74
Weight [kg]	77.7 (16.5)	79.2 (16.9)	0.55
BMI	26.3 (4.9)	26.9 (5.0)	0.43
Preoperative GCS	15 (13–15)	15 (5–15)	0.27
ASA status			0.39
1	13	9	
2	48	40	
3	33	39	
4	0	1	
n. d.^∗^	8	4	

^∗^n. d.: not determined (insufficient patient data).

**Table 2:** Patients laboratory baseline characteristics.

Parameter	Non-PFA group	PFA group	*P* value
Platelets [/nl]	257 (64)	254 (80)	0.72
Preoperative Hb [g/L]	144 (13.7)	143 (16.5)	0.72
Male	149 (12.2)	152 (13.8)	0.19
Female	139 (13.4)	136 (14.7)	0.28
Prothrombin time [INR]	0.99 (0.88–1.30)	0.98 (0.81–1.30)	0.19
aPTT [s]	28 (22–57)	28 (20–38)	0.33
Fibrinogen [g/L]	2.9 (1.0–8.0)	2.9 (1.5–7.0)	0.13
PFA-ADP [s]		99 (65–>300)	
PFA-Epi [s]	n. a.^∗^	107 (62–>300)	n. a.
Creatinine [mg/dL]	0.83 (0.21)	0.79 (0.21)	0.22

^∗^n. a.: not applicable.

**Table 3:** Distribution of primary diagnoses.

Diagnosis	Non-PFA group	PFA group	*P* value
Neuroepithelial tumors (e.g., astrocytomas and gliomas)	38	40	0.79
Meningeal tumors (e.g., meningiomas)	25	21	
Metastases	9	10	
Pituitary adenomas	7	7	
Others	23	15	

**Table 4:** Outcome data (continuous variables).

Parameter	Non-PFA group	PFA group	*P* value
Postoperative Hb [g/L]	116 (17.8)	116 (15.7)	0.90
Male	120 (17.7)	124 (14.6)	0.37
Female	111 (16.7)	110 (14.1)	0.87
Duration of anesthesia [min]	400 (159)	384 (131)	0.44
Duration of surgery [min]	294 (148)	281 (123)	0.51
Transfusions per patient			
Red cells [units]	0 (0–7)	0 (0–10)	0.39
Plasma [units]	0 (0–8)	0 (0–10)	0.91
Platelets [units]	0 (0–1)	0 (0–2)	0.33
Prothrombin complex [IU]	0	0	n. t.^∗^
Fibrinogen [g]	0 (0–4)	0 (0–3)	0.91
Postoperative GCS	15 (3–15)	15 (8–15)	0.69
GOS	5 (1–5)	5 (3–5)	0.10
LOS^∗∗^ in intensive care [days]	2 (0–33)	2 (0–43)	0.37

^∗^n. t.: not tested; ^∗∗^LOS: length of stay.

**Table 5:** Outcome data (categorical variables).

Parameter	Non-PFA group	PFA group	OR (95% CI)	*P* value
Transfused patients [*n*]				
Red cells	12	7	0.6 (0.2–1.6)	0.35
Plasma	17	16	1.0 (0.5–2.2)	1.0
Platelets	2	4	2.3 (0.4–12.6)	0.43
Fibrinogen	3	3	1.1 (0.2–5.6)	1.0
Patients receiving hemostatic treatment [*n*]				
Desmopressin	5	13	3.2 (1.1–9.2)	0.045
Aprotinin	2	5	2.8 (0.5–15.0)	0.14
Tranexamic acid	0	2	5.7 (0.3–119.6)	0.22
Clinical data [*n*]				
Intraoperative bleeding tendency	4	6	1.7 (0.5–6.2)	0.42
Clinically relevant bleeding complication	2	2	1.1 (0.4–2.8)	1.0
